# Supplementary material for: Methicillin-resistant Staphylococcus aureus along the beef production line: Phenotypic resistance and mecA phylogeny in two ethiopian municipal abattoirs
Source: PLoS One. 2026 May 7;21(5):e0334585. doi: 10.1371/journal.pone.0334585 (PMC13152186; doi:10.1371/journal.pone.0334585)

Antibiogram profile of the 38 *S. aureus* isolates

s.no	Sample Id	Doxycycline (DO30)	Norfloxacin (NX10)	Ceftazidime (CAZ30)	Penicillin (P10)	Clindamycin (CD2)	Methicillin (Met5)	Ampicillin (Amp10)	Erythromycin (E15)	Co-Trimoxazole (COT25	Gentamicin (CN10)	MDR
1	0Hb1	S	S	I	R	S	R	S	S	I	S	
2	0H3	R	S	I	R	R	R	R	I	S	S	**3**
3	0C20	R	S	R	R	R	R	R	I	S	S	**3**
4	0C7	R	S	I	R	S	R	I	S	S	S	
5	Oc4	R	S	S	R	R	R	R	I	S	S	**3**
6	1c1	R	R	R	R	R	R	R	R	R	S	**6**
7	1c4	S	S	S	R	S	R	S	S	S	S	
8	1cl1	R	S	I	R	S	R	R	I	S	S	
9	1C3	I	S	S	R	R	R	R	I	S	S	
10	1C21	R	S	R	R	R	R	R	I	I	S	**3**
11	1C22	I	S	S	R	S	R	S	I	S	S	
12	1C6	R	S	S	R	R	R	R	I	S	S	**3**
13	1H2	R	S	S	R	R	R	R	I	S	S	**3**
14	1W2	S	S	I	R	R	R	R	I	R	S	**3**
15	1Hb2	R	R	R	R	R	R	I	I	R	S	**5**
16	OW4	S	S	R	R	S	R	R	I	R	S	
17	OC23	R	S	R	R	S	R	S	I	S	S	
18	OW3	R	S	R	R	S	R	R	S	S	S	
19	OK6	S	S	R	R	R	R	R	I	S	S	
20	OHb4	R	S	R	R	R	R	S	I	R	S	**4**
21	OCL4	R	R	I	R	S	R	R	R	S	S	**4**
22	OA3	I	S	S	R	S	R	S	S	S	S	
23	OC8	R	S	I	R	R	R	R	S	s	S	**3**
24	OC9	I	S	S	R	R	R	S	I	R	S	**3**
25	OT1	R	S	R	R	R	R	R	I	I	S	**3**
26	OT3	R	S	S	R	R	R	S	I	R	S	**4**
27	OC15	R	S	S	R	S	R	S	S	S	S	
28	OHb5	R	S	I	R	R	R	R	S	S	S	**3**
29	1CL4	S	S	S	R	R	R	R	S	I	S	
30	1T1	R	S	R	R	R	R	R	I	S	S	**3**
31	1C25	R	S	R	R	S	R	R	R	S	S	**3**
32	1H6	S	R	S	R	R	R	S	I	S	S	**3**
33	1K5	S	S	R	R	R	R	S	I	R	S	**3**
34	1A5	R	S	I	R	S	R	S	S	R	S	**3**
35	1C15	R	S	I	R	R	R	R	I	R	S	**4**
36	1C19	S	S	S	R	S	R	S	I	S	S	
37	1K6	I	S	I	R	S	R	I	I	S	S	
38	1H7	R	R	R	R	R	R	R	S	S	S	**4**

I= Intermediate; R=Resistant; S=Susceptible

Antibiotics	Susceptible	Intermediate	Resistant
Doxycycline	9	5	24
Norfloxacin	33	0	5
Ceftazidime	13	11	14
Penicillin	0	0	38
Clindamycin (CD2)	15	0	23
Methicillin (Met5)	0	0	38
Ampicillin (Amp10)	13	3	22
Erythromycin (E15)	11	23	4
Co-Trimoxazole (COT25)	24	4	10
Gentamicin (CN10)	38	0	0

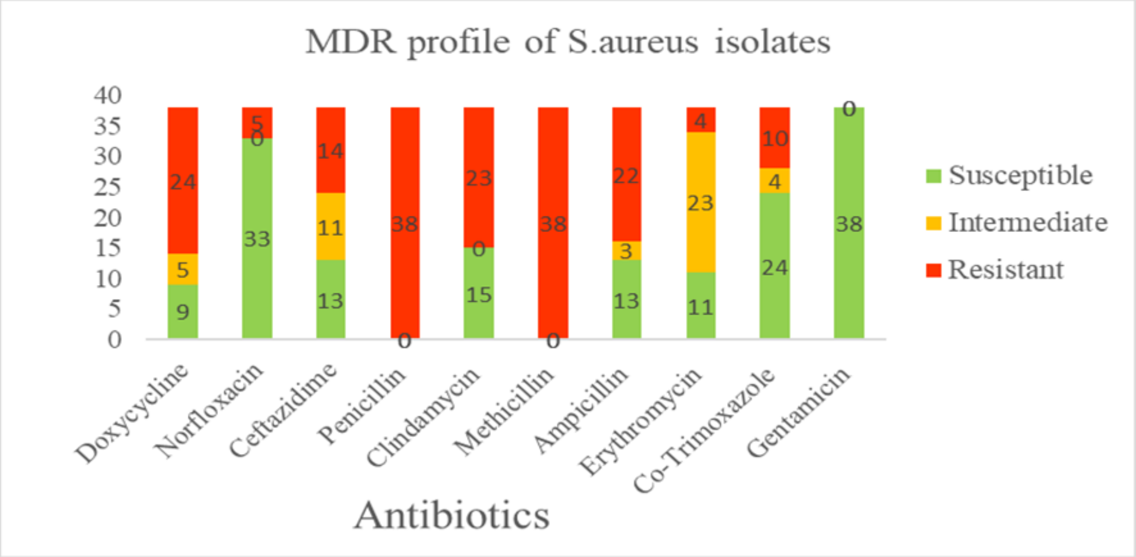

Supplement: S1 File — (PDF) [file pone.0334585.s001.pdf]
