# Supplementary material for: Methicillin-resistant Staphylococcus aureus along the beef production line: Phenotypic resistance and mecA phylogeny in two ethiopian municipal abattoirs
Source: PLoS One. 2026 May 7;21(5):e0334585. doi: 10.1371/journal.pone.0334585 (PMC13152186; doi:10.1371/journal.pone.0334585)
Supplement: S1 Fig — (PDF) [file pone.0334585.s002.pdf]

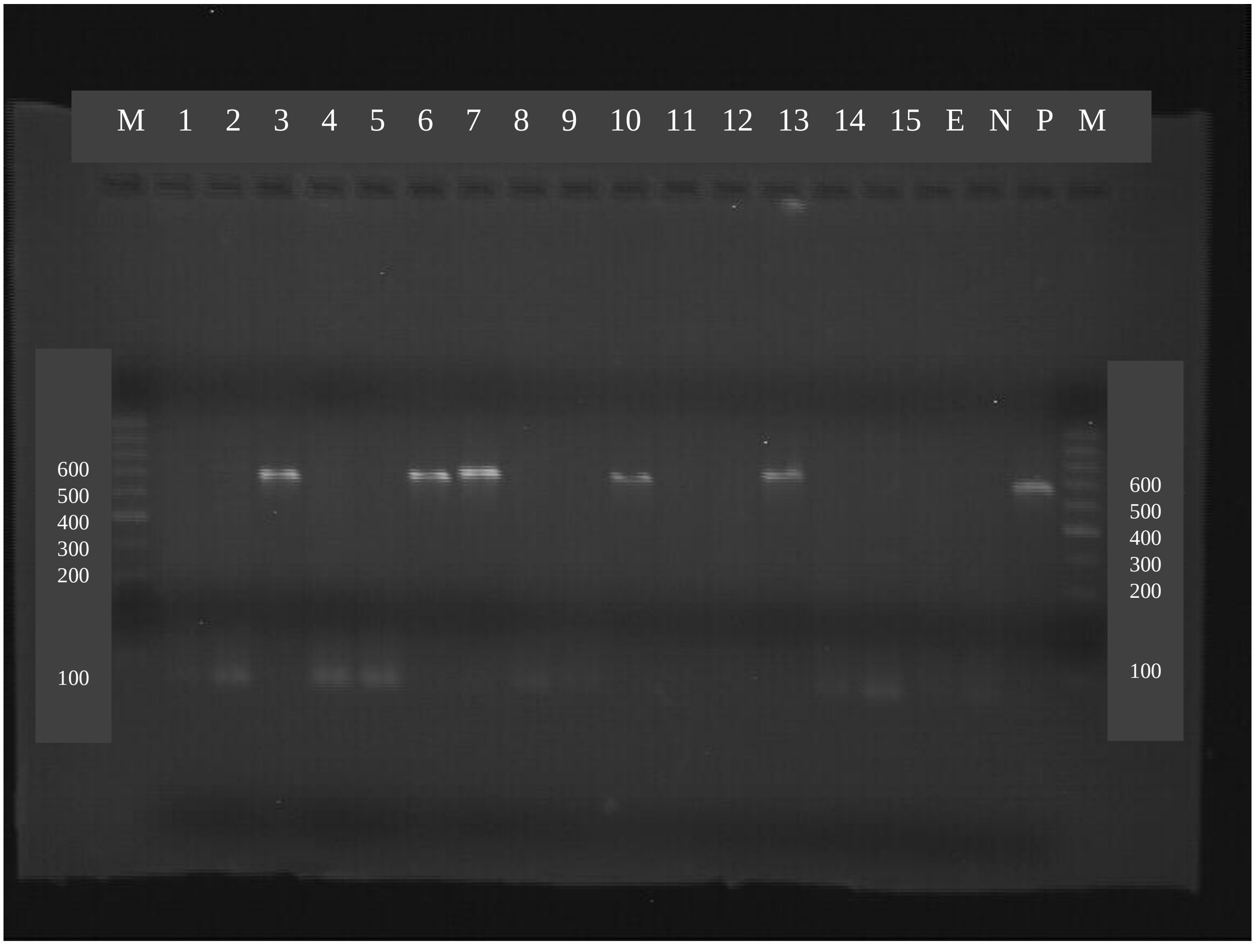


Agarose gel electrophoresis of *mecA* gene (533bp).

**M= DNA marker (100 bp); Lanes 1 – 15= test samples; E= extraction control; N= negative control, and P= positive control**
