# Supplementary material for: Methicillin-resistant Staphylococcus aureus along the beef production line: Phenotypic resistance and mecA phylogeny in two ethiopian municipal abattoirs
Source: PLoS One. 2026 May 7;21(5):e0334585. doi: 10.1371/journal.pone.0334585 (PMC13152186; doi:10.1371/journal.pone.0334585)
Supplement: Attachment — Submitted filename: Response to Reviewers_2.pdf [file pone.0334585.s003.pdf]

Response to Editor

Dear Editor-in-Chief,

Once again, we are thankful for the consideration of our manuscript in your journal and I have corrected the three enquires.

Sy. No.	Reviewers and/or Editor comments	Responses
Editor comments		
1.	We note that your Data Availability Statement is currently as follows: [All relevant data are within the manuscript and its Supporting Information files.] Please confirm at this time whether or not your submission contains all raw data required to replicate the results of your study.	Thank you very much for your request. All minimum required data and information are included in the manuscript and the Supporting Information files. Unfortunately, mean values and standard deviations are not available for this dataset. However, we have attached the file used to generate Fig 2, provided as “Supporting data_antibiogram profile.” In addition, the sequencing data for our samples are publicly available in GenBank under accession numbers PP735247 and PP735248.
2.	Please be informed that funding information should not appear in the Acknowledgments section or other areas of your manuscript.	I have removed any funding statement from the entire manuscript
3.	PLOS ONE now requires that authors provide the original uncropped and unadjusted images underlying all blot or gel results reported in a submission’s figures or Supporting Information files.	The uncropped gel image is included in the supporting information as a file name “S1_raw_images”
