# Supplementary material for: Methicillin-resistant Staphylococcus aureus along the beef production line: Phenotypic resistance and mecA phylogeny in two ethiopian municipal abattoirs
Source: PLoS One. 2026 May 7;21(5):e0334585. doi: 10.1371/journal.pone.0334585 (PMC13152186; doi:10.1371/journal.pone.0334585)
Supplement: Attachment — Submitted filename: Response to reviewers.pdf [file pone.0334585.s004.pdf]

Response to Reviewers

Dear Editor-in-Chief and Reviewers,

We are thankful for the consideration of our manuscript in your journal and for the constructive comments provided, which have significantly improved the quality and clarity of the manuscript. The full, detailed responses to all editorial and reviewer comments are presented below in table form.

Sy. No.	Reviewers and/or Editor comments	Responses
Editor and reviewer 2 comments		
1.	Lines 166–167: Reference 32 (Mehrotra et al., 2000) does not correspond to the same primers authors used for mecA amplification. The primers described in the manuscript differ from those in the cited reference. Please provide an appropriate and accurate reference.	Thank you for this important observation. We have carefully checked the cited reference and agree that it did not correspond to the primers used for mecA amplification in our study. Accordingly, we have replaced the previous citation (Mehrotra et al., 2000) with the appropriate reference (Murakami et al., 1991), which correctly describes the primer sequences used.
2.	Additionally, Please provide a clearer, high-resolution PCR gel image and verify the reported product size.	We did ourbest to get high resolution PCR ge image and the one we provided was the best. Additionally we provided un cropped PCR gel image as a separate file
3.	Lines 104–109: This section repeats information presented in the following paragraph. Please remove the redundant text to improve clarity and avoid duplication.	Thank you for the comment and removed based on the recommendation
4.	Line 136: The term "Gram" should be written in lowercase (i.e., "gram").	Thank you and corrected based on the recommendation
5.	Lines 144–146: Please group antibiotics according to their respective classes and clearly mention the class names. Additionally, ensure consistency in formatting—antibiotic names should follow a uniform style throughout the manuscript (e.g., consistent capitalization).	Thank you for this helpful comment. The antibiotics listed have been revised and grouped according to their respective classes, with class names clearly indicated. In addition, the formatting of antibiotic names has been standardized throughout the manuscript to ensure consistent capitalization and style.
6.	Line 152: Scientific names should be written in italics.	The funding information is only appear in funding statement section

7.	Line 160: The sentence is unclear. It is recommended to revise it as: "Genomic DNA was extracted from Staphylococcus aureus."	Thank you for the helpful suggestion. The sentence in has been revised for clarity as recommended.
8.	Line 183: The DNA extraction method has already been described earlier. Repetition is unnecessary—please remove this section.	Thank you for this valuable comment. To avoid unnecessary repetition, the repeated description has been removed from the revised manuscript.
9.	Line 250: Scientific names should be written in italics.	Thank you and corrected based on the recommendation
10	Line 251: The gene name mecA is inconsistently formatted (italicized in some places and not in others). Please adopt a consistent style throughout the manuscript.	Thank you for this important observation. We have carefully reviewed the manuscript and corrected the formatting of the mecA gene name to ensure consistency throughout the text. All instances have now been uniformly formatted in italics as recommended.
Reviewer #1 comments		
1.	The grammar needs a bit improvement. The S. aureus should be uniformly italic across the article.	Thank you for this helpful comment. The manuscript has been carefully revised to improve grammar throughout the text. In addition, the formatting of Staphylococcus aureus (S. aureus) has been standardized and is now consistently italicized across the entire manuscript, as recommended.
Reviewer #2 comments		
All comments are the same with editor and please refer to the responses provided in the editor section above		
